# Surgical Cases in the Private Practice of Prof. Julius F. Miner

**Published:** 1878-08

**Authors:** Charles A. Wall


*ART. II.—Surgioal Cases* in the private practice of Prof. JULIUS F. MINER, M. D. Reported by Charles A. Wall, B. S.

CASE I. Mrs. M., æt. 45; widow; American. The patient who had borne two children, both now living, was a healthy woman until six years previously, when a small tumor made itself manifest in the left inguinal region, which seemed to attach itself to the uterus. Its growth was moderately troublesome until about the first of January, 1877, from which time it increased rapidly. She was examined by a number of physicians, all of whom refused to operate, fearing adhesions and attachments to the uterus. June 6, 1877, after careful examination, Dr. Miner determined there were grounds to expect the tumor might be removed with usual safety. Ether was used to produce anæsthesia, which was taken pleasantly. An exploratory incision having been made there was found to be a multilocular ovarian tumor which was removed in the usual manner, but as the pedicle was found to be very short and large vessels extending into the solid portions of the tumor direct without subdivision, a double ligature was passed through the pedicle as near its end as possible and tied. The tumor was severed and the pedicle stitched into the lower angle of the wound. The incision was closed with interrupted sutures, warm water dressings applied and the patient put to bed. The tumor weighed with contents about thirty pounds, emptied of its contents it weighed twelve. It consisted of one large cyst, a few moderate sized, and many small cysts with a solid portion weighing about seven pounds. The pedicle was attached to the solid portions, many of the arteries entering the tumor direct and about the size of a small quill, they then subdivided and supplied the partitions between the various cavities. The pedicle was noticable from its shortness and thickness, it was the fallopian tube enlarged and when it was stitched into the angle of the wound it tilted the uterus up and turned its lateral diameter nearly anterio-posteriorly. She rallied well from the ether, and at 6 P. M. the same day her pulse was 84, temperature normal. She continued improving until June 20th, when by excitement from visiting with her son and writing a letter, unknown to the nurse, her pulse went up to 120, temperature 104, these came down in a day or two and she went on to a good recovery. She did not evacuate her urine naturally without the use of the catheter for nearly three weeks, after which she had no more trouble. The unnatural strained position of the uterus has not been a source of discomfort, which is the most remarkable feature of the case, the distance from the abdominal parietes to the uterus not being over an inch.

Case II. *Ascites, Paracentesis Abdominalis,* recovery. Mrs. S., æt. 21, American, had been married about a year when she began to be troubled with an increase in the size of the abdomen, pregnancy was suspected but found to be absent, and upon examination July 9th, Dr. Miner diagnosed ascites and tapped it, drawing off about ten quarts of a clear limpid fluid which would not coagulate upon the application of heat. In every other respect she seemed healthy, and no cause could be found for the accumulation of the fluid. The kidneys were normal in their action as were the other organs, so far as could be ascertained.

July 10th, 1878. There has been no recurrence of the trouble and she has been uniformly healthy. Nothing has as yet shown itself to indicate the original cause of the ascites.

Case III. *Polypus on neck of Uterus.* Mrs. P., æt. 60, has long suffered from an offensive discharge from the vagina, which had by its corroding action made the external genitals tough, hard and inelastic as leather. Astringent washes were used for some time with little effect, except to reduce the excessive tenderness of the parts. July 15th, 1877, upon examination by speculum, a small polypoid growth upon the anterior lip of the os uteri was discovered. This was taken away with a pair of forceps, and chromic acid used to cauterize its base which seemed to remove the trouble, and the discharge from the vagina soon ceased. In the course of a few months the parts took on a healthy nature and she has not suffered with any return. The case is peculiar in that so small a polypus (not larger than a bean,) could create so much disturbance. The external parts were thickened, enlarged and toughened almost past recognition; they seemed more like leather than healthy human skin. The vagina was inflamed and excessively tender. The result was astonishing, soon after the polypus was removed the discharge ceased and the parts began to change and grow more healthy, and it was only a few weeks before they returned to their natural color and flexibility.

Case IV. *Artesia Vagina.* Mrs. R., æt. 30, German. Two years previously had long tedious labor, in which forceps were used followed by ulceration of the parts which occluded the vagina, the occlusion was so perfect that the smallest probe could not be introduced. Anæsthesia having been induced, operation was made July 20th, 1877. The parts were separated until the finger could be passed into the vagina. The cicatrix was then carefully cut out and the parts brought together by interrupted sutures. This necessitated taking out a piece extending nearly around the vagina; in front it was only about one-quarter of an inch, while at the posterior surface the width was nearly an inch. Care was taken to remove all the cicatricial tissue and bring the mucous membrane into approximation. In no place were the deep tissues invaded, and as the wound healed by first intention no further operative measures were needed. In two weeks she was well and a medium sized speculum could be introduced. The parts were healthy and normal, while the line of union could not be made out. This is a rare case from the closeness of the occlusion and the ease with which it was removed.

